# Correction: Most Undirected Random Graphs Are Amplifiers of Selection for Birth-Death Dynamics, but Suppressors of Selection for Death-Birth Dynamics

**DOI:** 10.1371/journal.pcbi.1007143

**Published:** 2019-06-14

**Authors:** The PLOS Computational Biology Staff

## Notice of republication

This article was republished on November 24, 2015, to correct errors in the Supporting Information. The publisher apologizes for the errors. Please download this article again to view the correct version.

## Supporting information

S1 File Republished, corrected article. (PDF)
