# Supplementary material for: Most Undirected Random Graphs Are Amplifiers of Selection for Birth-Death Dynamics, but Suppressors of Selection for Death-Birth Dynamics
Source: PLoS Comput Biol. 2015 Nov 6;11(11):e1004437. doi: 10.1371/journal.pcbi.1004437 (PMC4636432; doi:10.1371/journal.pcbi.1004437)
Supplement: S1 Text — (PDF) [file pcbi.1004437.s001.pdf]

Proof for Eq. (15)

Here, we prove that a mutant on the cycle with dB updating has lower fixation probability than in the well-mixed population for general N. We have to show that $\phi_{dB}^M - \phi_{dB}^\circ > 0$ for $r > 0, r \neq 1$. Under dB updating, the fixation probability in the well-mixed population is given by

$$\phi_{dB}^M = \frac{N-1}{N} \frac{1-\frac{1}{r}}{1-\frac{1}{r^{N-1}}}. \qquad (1)$$

On the cycle, the respective fixation probability is given by (Eq. (5.3) in [1])

$$\phi_{dB}^\circ = \frac{2(r-1)}{3r-1+(r-3)r^{2-N}}. \qquad (2)$$

Then the difference is given by

$$\phi_{dB}^M - \phi_{dB}^\circ = \frac{(r-1)r^{N-2}}{N} \cdot \frac{(N-3)r^{N+1} - (N-1)r^N + (N-1)r^3 - (N-3)r^2}{(r^{N-1}-1)(3r^{N+1} - r^N + r^3 - 3r^2)} \qquad (3)$$

This expression can be written in the form of

$$\frac{(r-1)^2 r^{N-2}}{N} \cdot \frac{\sum_{k=0}^{N-4} r^k((N-2)(k+1)-(k+1)^2)}{\left(\sum_{k=0}^{N-2} r^k\right)\left(3+\sum\limits_{k=1}^{N-3} 2r^k + 3r^{N-2}\right)}. \qquad (4)$$

As Eq. (4) contains only positive coefficients in r, the difference is always positive for $r > 0$ and $r \neq 1$. For $r = 1$, it is zero. Thus Eq. (15) is fulfilled for all $r > 0$, $r \neq 1$.

References

[1] Kaveh K, Komarova NL, Kohandel M. The duality of spatial death-birth and birth-death processes and limitations of the isothermal theorem. Journal of the Royal Society Open Science. 2015;2(140465).
